# Determinants of patient participation for safer care: A qualitative study of physicians' experiences and perceptions

**DOI:** 10.1002/hsr2.87

**Published:** 2018-09-12

**Authors:** Kristina Schildmeijer, Per Nilsen, Carin Ericsson, Anders Broström, Janna Skagerström

**Affiliations:** ^1^ Department of Health and Caring Sciences Linnaeus University Kalmar Sweden; ^2^ Department of Medical and Health Sciences Linköping University Linköping Sweden; ^3^ Cardiology and Speciality Medicine Centre University Hospital in Linköping, Region Östergötland Sweden; ^4^ Department of Nursing, School of Health and Welfare Jönköping University Jönköping Sweden; ^5^ Research and Development Unit in Region Östergötland Linköping Sweden

**Keywords:** barriers, determinants, facilitators, patient participation, patient safety, physicians

## Abstract

**Objective:**

There is a paucity of research on physicians' perspectives on involving patients to achieve safer care. This study aims to explore determinants of patient participation for safer care, according to physicians in Swedish health care.

**Methods:**

We used a deductive descriptive design, applying qualitative content analysis based on the Capability‐Opportunity‐Motivation‐Behaviour framework. Semi‐structured interviews were conducted with 13 physicians in different types of health care units, to achieve a heterogeneous sample. The main outcome measure was barriers and facilitators to patient participation of potential relevance for patient safety.

**Results:**

Analysis of the data yielded 14 determinants (ie, subcategories) functioning as barriers and/or facilitators to patient participation of potential relevance for patient safety. These determinants were mapped to five categories: physicians' capability to involve patients in their care; patients' capability to become involved in their care, as perceived by the physicians; physicians' opportunity to achieve patient participation in their care; physicians' motivation to involve patients in their care; and patients' motivation to become involved in their care, as perceived by the physicians.

**Conclusion:**

There are many barriers to patient participation to achieve safer care. There are also facilitators, but these tend to depend on initiatives of individual physicians and patients, because organizational‐level support may be lacking. Many of the determinants are interdependent, with physicians' perceived time constraints influencing other barriers.

## 1 INTRODUCTION

Patient participation to achieve increased patient safety has become an area of increasing interest in policy making, research and health care management, and practice. Patient participation can refer to patients' participation in the decision‐making process regarding various health issues, but the concept is usually understood in broader terms as patients participating in “many and varied aspects” of health care.^1^ Patients are uniquely placed to observe their care and physical environment throughout their journey in the health care system.^2^ Patient participation has been shown to improve decision making and treatment of chronic diseases, eg, educational interventions for self‐management and prompts given to patients to perform specific tasks.^1^ It is reasonable to believe that it could also help prevent errors, although there is not much research to support this conclusion.

Findings concerning patients' willingness and ability to participate in their own care are somewhat inconsistent. Research has shown that patients are not always prepared to commit time and energy to improve their care because they have enough to worry about when they are ill.^3^, ^4^ For many patients, physicians and other health professionals represent traditional medical authorities, and questioning them is an unacceptable extension of the patient's role.^4^ Studies show that patients may be apprehensive about reporting problems within their care when they interpret practitioners' responses as unappreciative or when the patients believe that their feedback may jeopardize the practitioners' goodwill towards the patient.^1^, ^3^, ^5^

Physicians can potentially play an important role in engaging patients to achieve safer care by exchanging information, building a good interpersonal trustful relationship, and sharing decision making.^6^, ^7^, ^8^ However, there is a lack of empirical research on how or the extent to which this potential is realized.

We have identified a few studies^1^, ^9^, ^10^, ^11^ that have addressed physicians' perspectives on patient involvement for safer care; two studies^9^, ^12^ have used hypothetical hand hygiene and medication error scenarios. Schwappach et al^12^ conducted a study in Switzerland involving 1141 health care professionals (15% of which were physicians). They found that the professionals, in general, approved of the patient intervening, eg, reminding professionals to wash their hands, even if they believed this could have negative effects on the physician‐patient relationship. In a study carried out by Davis et al^9^ in the UK, involving 216 health care professionals (of which 54% were physicians), they found that the professionals perceived patients who asked about a potential error less favourably if the patient's behaviour was considered confrontational in nature, than if the enquiry was polite.

Despite increased attention to patient participation in health care, there is a paucity of research on physicians' perspectives on involving patients to achieve safer care. In response to this knowledge gap, this study aims to explore determinants of patient participation for safer care, according to physicians in Swedish health care. Improved understanding of the factors that influence how physicians can involve patients in their care is important for physicians and other health professionals, patients, decision makers, and policy makers to achieve improved patient safety.

## 2 METHODS

### 2.1 Study setting

The study was carried out in Sweden. Health care in Sweden is mainly publicly funded, although private health care also exists. All residents are insured by the state, with equal access for the entire population. Out‐of‐pocket fees are low and regulated by law. The provision of health care services in Sweden is primarily the responsibility of the 21 county councils throughout Sweden. The health care system is financed mainly through taxes levied by county councils and municipalities.

### 2.2 Study design and participants

We used a deductive descriptive design with qualitative content analysis, based on a framework called Capability‐Opportunity‐Motivation‐Behaviour (COM‐B),^13^ described below.

Thirteen semi‐structured interviews with physicians were conducted. We used a purposive sampling strategy to achieve a broad range of perspectives from the target population of interest, with regard to working in different health care units and with patients who varied in terms of general health status (from patients seen in primary health care to inpatients in hospital care), health condition (from acute to chronic diseases and illnesses), and duration of health care contact (from outpatients to inpatients). The aim was to achieve a sample of physicians that represented a broad spectrum of experiences and perceptions concerning patient participation in relation to patient safety. A sample of 10 to 15 physicians was considered to be appropriate as the objective was narrow, and the population homogeneous and in possession of a high degree of expertise about the interview subject.^14^, ^15^

The physicians were employed in several different health care units, all located in cities (with 67 000, 135 000, and 150 000 inhabitants) in south‐east Sweden: a pulmonary ward in a university hospital (540 beds); a surgery unit in a mid‐sized hospital (350 beds); an ear nose and throat unit in a mid‐sized hospital (500 beds); a rheumatology department in a university hospital (540 beds); and two primary health care centres.

The physicians were recruited via an e‐mail that briefly described the study. The e‐mail request was sent to the manager of each health care facility. The manager, in turn, forwarded the request to all physicians at the unit. Physicians who were interested in participating contacted the researchers directly. An information letter describing the study aim and procedure of the interview was sent to interested physicians, and the interviews were scheduled. No respondents declined involvement after receiving the information letter. All respondents provided written informed consent to participate in the study.

### 2.3 Data collection

An interview guide with qualitative semi‐structured questions was used for data collection.^16^ The questions were generated by the research team with reference to the existing literature on patient participation for safer care.

The questions were tested in one pilot interview with regard to meaningfulness for participants and clarity of concepts. The pilot interview indicated that the wording was clear and that the interview did not exceed 45 minutes (deemed feasible with regard to the participants' busy work schedule).

The interview guide (see Supporting Information) dealt with issues concerning the physicians' experiences and perceptions regarding patient participation relevant to patient safety. There were general questions on how patients can influence patient safety, as well as more specific questions concerning their own experiences and examples of patients who have observed something of importance for patient safety. The interview guide ended with questions on existing routines and tools to account for patients' views, experiences, and suggestions on how patient participation to reduce errors in health care can be achieved.

During the interviews, the interviewer asked probing questions, eg, “Can you explain a little further?”. Towards the ends of the interviews, the interviewer asked if there was more to discuss or if something needed further clarification. All interviews were recorded and transcribed verbatim.

The interviews took place between June 2015 and June 2016, K.S., C.E., and J.S., conducted and recorded the interviews. Transliteration was made by a professional company. No extra compensation was given to the physicians because the interviews were held during regular working hours at the health care units where the participants worked. No physician had a relationship with any of the researchers. The interviews lasted between 18 and 40 minutes (mean, 29 minutes).

### 2.4 Theoretical framework

We applied the COM‐B framework developed by Michie et al^13^ to categorize the barriers and facilitators (ie, determinants) to patient participation of potential relevance to patient safety. Barriers were defined as physicians' and patients' actions, beliefs, and attitudes that made patient participation for safer care more difficult, whereas facilitators were defined as physicians' and patients' actions, beliefs, and attitudes that made patient participation in safer care easier. Some determinants could function as both barriers and facilitators.

Capability‐Opportunity‐Motivation‐Behaviour sets out that behaviour, eg, a physician's communication with a patient, comes about from an interaction between the capability to perform the behaviour and the opportunity and motivation to carry out the behaviour. The framework is intended to be comprehensive, parsimonious, and applicable to all behaviours. Earlier studies have applied COM‐B to many different types of behaviours, including patients' medication adherence,^17^ audiologists' behavioural planning,^18^ and health coaching for women with diabetes.^19^

Capability is defined as an individual's psychological and physical capacity to engage in the behaviour. It includes having the necessary knowledge, ability, and skills. Opportunity is defined as all the factors that lie outside the individuals that make the behaviour possible or prompt it, eg, the physical environment, and the social, and cultural context. Motivation or willingness to enact behaviour is defined as all those brain processes that energize and direct individuals' behaviour, including habitual processes and emotional responding, as well as analytical decision making.^13^

### 2.5 Data analysis

The interview data were analysed using directed content analysis in accordance with Hsieh and Shannon.^20^ A directed approach was deemed applicable because we wanted to analyse determinants of patient participation for safer care, as perceived by the physicians who were interviewed, using the categories of an existing framework, ie, COM‐B.

As a first step, all the authors read all transcripts to obtain an understanding of the whole. The transcripts were then coded separately by two of the authors, K.S. and P.N., using a directed content analysis that included a structured analysis process to code and categorize the data using COM‐B. Hence, COM‐B was used to determine the coding and relationships between the codes. Data that could not be coded or identified in relation to the framework in the first step were analysed later to determine if the data represented a new category or a subcategory of an existing category.^20^

In the next stage, K.S. and P.N. discussed the interpretation of the data in relation to COM‐B and compared their coding. They then presented their categories (ie, capability, opportunity, and motivation) and their contents to the other researchers. Discussions in the group continued until no inconsistencies existed and a shared understanding was reached to increase trustworthiness and strengthen the internal validity.^21^ Representative quotations were identified to report the findings. Quotations were then translated from Swedish to English by P.N. and K.S., both of whom have a sufficient degree of expertise in the English language to supervise this process. No back translation was done. The physicians are numbered 1 to 13 in the Results section.

### 2.6 Ethical considerations

The study was performed according to the World Medical Association Declaration of Helsinki ethical principles for medical research involving human subjects. The project followed the recommendations of the Central Ethical Review Board of Sweden concerning information to the research participants on actions to ascertain confidentiality and voluntariness of the interviews. An application for handling personal data was approved (according to The Swedish Data Protection Authority). The study did not require ethical approval because it did not involve sensitive personal information, as specified in Swedish law regulating ethical approval for research concerning humans.^22^

The health care managers forwarded the invitation to all physicians at their clinic and those who were interested in participating contacted us by email or telephone. All the participants gave their written informed consent to participate in the interviews. The researchers did not have any further contact with the managers after that the invitation to the study was forwarded to the physicians. In the information letter, it was stated that the physicians could stop the interview at any time (but no one chose to do so). The physicians could also decide the time of the interview themselves, if, for example, they did not want their colleagues or managers to know that they have been interviewed. If any present misconduct would have been disclosed, the authors had decided to recommend the physician to report it according to regular routines. To ensure confidentiality, all sound files as well as transcripts were stored on computers requiring log in. No lists of names or work units were kept, and data were not reported at a personal level.

## 3 RESULTS

Thirteen physicians were interviewed for the study, of which seven were male and six were female. Eleven of the 13 had worked as a physician for more than 10 years. The median number of years working in the health care facility in which they were recruited was 7 years (Table 1).

**Table 1:** Participant characteristics

Characteristics	Physicians (*N* = 13)
Sex	
Male	7 (53.8)
Female	6 (46.2)
Years of practice	
0‐1 years	1 (7.7)
2‐4 years	0 (0)
5‐9 years	1 (7.7)
10‐20 years	7 (53.8)
21 years or more	4 (30.8)
Median years of practice (range)	16 (1‐38)
Years in the health care facility	
0‐1 years	2 (15.4)
2‐4 years	2 (15.4)
5‐9 years	4 (30.8)
10‐20 years	5 (38.5)
21 years or more	0 (0)
Median years in the health care facility (range)	7 (0.5‐15)
Health care facility	
Pulmonary medicine ward	3 (23.1)
Surgical ward	2 (15.4)
Ear, nose and throat clinic	2 (15.4)
Rheumatology department	3 (23.1)
Two primary health care centres	3 (23.1)

Values are number (% of total) except where indicated.

Analysis of the data yielded 14 determinants (ie, subcategories) functioning as barriers (B) and/or facilitators (F) to patient participation of potential relevance for patient safety (Table 2). These 14 determinants were mapped to five types of major determinants (ie, categories) of patient participation that could contribute to improved patient safety: physicians' capability to involve patients in their care (C in COM‐B); patients' capability to become involved in their care, as perceived by the physicians (C); physicians' opportunity to achieve patient participation in their care (O); physicians' motivation to involve patients in their care (M); and patients' motivation to become involved in their care, as perceived by the physicians (M).

**Table 2:** Classification of the determinants identified in the data

Category in the COM‐B Framework	Type of Determinant (Categories)	Determinants (Subcategories)
Capability	Physicians' capability to involve patients	Trust in the patient (B + F) Communication with the patient (B + F) Attention to the patient (B)
Capability	Patients' capability to become involved	Patients' physical and mental health status (B) Patients' knowledge and understanding (B) Patients' cognitive overload of information (B)
Opportunity	Physicians' opportunity to achieve patient participation	Time (B) Continuity of care (B + F) Routines and tools (B + F)
Motivation	Physicians' motivation to involve patients	Initiating patient engagement (B + F) Learning from patients who detect errors and safety hazards (B + F)
Motivation	Patients' motivation to become involved	Patients' perceptions of physician authority (B) Patients' communication of sensitive information (B) Patients' socio‐demographic characteristics (B + F)

B, determinants that acted as barriers; F, determinants that acted as facilitators; B + F, determinants that could function both as barriers and facilitators.

### 3.1 Physicians' capability to involve patients in their care

#### 3.1.1 Trust in the patient (B + F)

Physicians' capability to interact with patients constituted a determinant of patient participation of potential relevance to patient safety. Failure to establish such trust could result in missed opportunities to capture safety hazards related to the patient's treatment or care that the patient may detect. On the other hand, physicians also described how a satisfactory degree of trust could facilitate patient participation. “The most important thing is that you [the patient] can be open and say what's in your heart, and that requires a degree of trust that makes it possible to do this” (Participant IX).

#### 3.1.2 Communication with the patient (B + F)

Physicians' communication with the patient, including various forms of information, was another determinant of patient participation. Examples included physicians asking patients for confirmation of understanding the information provided and repeating important information. “If you [the patient] have good information, there are many things you won't need to discuss or ask about because it has already been clarified” (Participant XI).

Physicians also described instances where communication could function as a barrier to patient participation. According to physicians, communication that is too standardized and not sufficiently adapted to each patient's unique understanding or capacity could lead to missed opportunities to identify safety hazards via the patient. “If you talk about patient safety and the patient's own involvement, it's important that the patient is ‘with you’ all the way, that you work from this patient's existing knowledge, capacity, wishes, and desires. And we don't individualize information that way” (Participant I).

#### 3.1.3 Attention to the patient (B)

Further, physicians stated that insufficient attention, eg, due to meetings or treating large numbers of patients, could cause them to overlook safety hazards that the patient may hint at or bring up in conversation. “You do it [surgical operations] many times, and the focus tends to wander so you don't really notice things; you get a bit speed‐blinded” (Participant V).

### 3.2 Patients' capability to become involved in their care, according to the physicians

#### 3.2.1 Patients' physical and mental health status (B)

Physicians mentioned that patients' physical or mental illnesses or limitations could restrain their ability to become involved in their own care. “There are also patients who cannot make their voice heard or who are intellectually disabled, so they do not intellectually grasp why they're controlled, and that means bigger risks” (Participant XII).

#### 3.2.2 Patients' knowledge and understanding (B)

Physicians' expressed that issues with patients having limited understanding to detect errors and safety hazards with regard to various aspects of their treatment, care, or health care environment constituted a barrier to patient participation. Physicians argued that many patients are simply not sufficiently cognizant concerning their illness or care to be able to make meaningful contributions to safer care. One physician remarked that “we are experts in what we do, but the patient is like a beginner in our environment” (Participant III). Another mentioned that “How should the patient understand what risks are unnecessary and could have been avoided?” (Participant V), because patients may have insufficient knowledge and understanding.

Further, communication barriers could hinder patient participation for safer care, according to the physicians. “There can be a language barrier. Sometimes they [the patients] don't understand the symptoms; they don't understand. Well, then it [the information] can fall between the cracks and something will go wrong” (Participant X).

#### 3.2.3 Patients' cognitive overload of information (B)

Physicians further contended that patient participation could depend on how patients reacted to the situation they were in. Patients who are unaccustomed to the health care environment may experience a sort of sensory “overload” because they receive information from many sources and meet with numerous practitioners. These circumstances might make it difficult to be potentially active in one's own treatment or care, thus acting as a barrier to patient participation, especially for those patients who have multiple illnesses. “There are many things going on at once, several illnesses, several different health care providers, and many medications. A lot of information, to sort out and combine” (Participant X).

### 3.3 Physicians' opportunity to achieve patient participation for safer care

#### 3.3.1 Time (B)

The physicians frequently brought up time constraints as a barrier to patient participation of potential relevance to patient safety. They recognized that shortage of time with their patients makes it difficult to establish trust with the patients and address all questions and concerns a patient might have. “We don't always have the time to give the patient the attention he believes he should have or thinks he deserves” (Participant XI). Shortage of time can lead to stress. “It's a question of time. Patients can have a lot of opinions, and there can be many opinions that are valuable, but there are also opinions that you do not feel you have the possibility or time to take care of, at the moment. And then this stress factor enters, which is a big safety hazard in health care” (Participant I). Physicians also noted that their time with patients was not always spent wisely. “Sometimes we go into discussions about things that aren't so relevant. That's not good for the patient, as the focus turns to something that might not be so important, and it takes a lot of time” (Participant VIII).

#### 3.3.2 Continuity of care (B + F)

Physician continuity influenced the opportunity to achieve patient participation. “In many places, you might meet a new health care provider every time, someone who is a stranger, and I believe continuity facilitates the possibility to criticize” (Participant IX). Whereas lack of or poor continuity constituted a barrier, physicians believed that good continuity could act as a facilitator because it could generate a more trustful relationship with patients. “I think it would make it easier if we had better continuity in health care, so you met the same person every time. … It would make it easier to convey both positive and negative critique” (Participant X).

#### 3.3.3 Routines and tools (B + F)

Routines and tools to provide support or more hands‐on guidance for achieving patient participation constituted another determinant of involving patients. Physicians commented on how the lack of recommendations concerning how to involve patients in their care can act as a barrier. “There is nothing standardized that tells you that you should ask the patients what they have understood of this” (Participant I). In general, the physicians did not identify any existing routines or tools that facilitated patient participation. However, they described numerous activities that involved patient participation to some degree, eg, a national patient survey questionnaire and various forms of adverse events reporting. They had ideas for further tools to achieve improved patient participation of potential relevance to patient safety, including providing new patients with an introductory leaflet encouraging them to voice their opinions and ask questions, provision of a brief patient questionnaire directly related to delivery of care, a discharge dialogue to account for patient complaints and concerns, and some form of “talk‐back” technique to make sure the patient has understood and processed all the information.

### 3.4 Physicians' motivation to involve patients in their care

#### 3.4.1 Initiating patient engagement (B + F)

Physicians noted the challenges of initiating patient engagement, which appeared to function as a barrier or facilitator, depending on the physicians' actions. They recognized the importance of actively inviting the patient to communicate; one physician noted that “if you're not invited to the conversation, it's difficult” (Participant I). However, physicians with previous experience with patients who never seemed to be content, expressed reluctant feelings to actively engaging patients. “Sometimes you think when a patient has started asking a lot of questions and they have received many replies, that they should be satisfied” (Participant VI).

#### 3.4.2 Learning from patients who detect errors and safety hazards (B + F)

Physicians expressed ambivalence about patients who detected slips or mistakes in their clinical practice. Learning from such patients seemed to function as a barrier or facilitator to patient participation, depending on the individual physician. One physician argued that “it has to do with your personality and how well you can handle criticism” (Participant VII). Another physician observed that “it's possible that some [physicians] think it's a distrust of their professionalism or that they [patients] step over some sort of boundary that makes them feel attacked” (Participant I). The physicians were not entirely comfortable with patients who pointed out errors or safety hazards that they were unaware of. “It can be embarrassing, humiliating, if you recognize you have made a mistake” (Participant II). At the same time, physicians also expressed sentiments of gratitude to inquisitive patients. Physicians mentioned that patients who asked many questions could make them think twice. “If it's me who has made the mistake, it may feel awkward, so, but it's great that they say it” (Participant VI). They also said that there were situations when the patients knew things about their medication or health that were unknown to the physicians. Some physicians stated that they were relieved that patients had observed errors and hazards before something undesirable occurred.

### 3.5 Patients' motivation to become involved in their care, according to the physicians

#### 3.5.1 Patients' perceptions of physician authority (B)

Physicians recognized patients' reluctance to question physicians' authority as a barrier to patient participation. They argued that this hesitancy could lead to patients refraining from asking pertinent questions or commenting on circumstances to prevent errors. “The patient is usually in some kind of subservient position. The doctor has a certain authority and you don't dare to question. They [the patients] are also at a knowledge deficit” (Participant X).

#### 3.5.2 Patients' communication of sensitive information (B)

Physicians said that safety hazards may go undetected when patients are unwilling to communicate information that the patient may consider to be sensitive. “There might be things you [the patient] are ashamed of saying, which you think are not important. You might worry that mentioning this might have undesirable consequences” (Participant X).

#### 3.5.3 Patients' socio‐demographic characteristics (B + F)

Physicians provided anecdotal evidence that suggested that patients' motivation to participate was associated with various socio‐demographic characteristics, such as age, level of education, and social and cultural background. There appeared to be few consistent patterns with regard to how the various characteristics affected patient participation. Older age was an exception; it was viewed by many physicians as a barrier to patients' motivation to participate in their care. Some physicians commented that younger patients tend to be more inquisitive than older patients, thus being a facilitator for patient participation. “The younger generation is quite different. I mean, they are demanding in a different way” (Participant IV).

## 4 DISCUSSION

This study explored determinants of patient participation to achieve safer care, as perceived by physicians in Swedish health care. We decided to investigate the views of the health care professionals and patients in separate studies to allow for a more in‐depth and comprehensive description of both perspectives (a study from the patient perspective is currently being undertaken by the research team behind this study). Based on the physicians' accounts, we identified 14 determinants (barriers and/or facilitators). These were mapped onto the COM‐B framework.^13^ We found the structure and content of COM‐B to be relevant for the study, offering a useful framework for organizing determinants of patient participation of potential relevance for patient safety and to categorize facilitators and barriers. We did not identify any determinants that did not fit into the framework. Eight of the determinants functioned solely as barriers to patient participation, and seven could act as barriers or facilitators, depending on the individual physicians and patients involved. No determinants functioned solely as facilitators, which could reflect that the physicians find patient participation difficult. It may also be because physicians instinctively think of barriers when they discuss this topic because they are used to critical thinking.

The physicians believed that patients' motivation to be involved in their own treatment and care was constrained by the patients' impression of being subordinate to physicians, whom they recognized as an authority that they were reluctant to question or criticize. The physicians seemed very much aware of patients' respectful views of them. Our findings are consistent with previous research conducted from the point of view of patients, which found that patients believe it is inappropriate to challenge health professionals in general,^23^, ^24^ are fearful of questioning medical authority,^25^, ^26^ and are afraid of offending physicians.^27^, ^28^, ^29^, ^30^ It has been shown that patients consider that physicians hold an elite position in health care; thus, they are more willing to ask challenging safety‐related questions to nurses, and factual questions related to quality and safety, to physicians.^28^

The time‐pressed everyday clinical practice of most physicians in this study presented a barrier to patient participation for safer care. Again, this finding is very much consistent with previous research, which has found that a busy clinical setting inhibits patients' involvement in their treatment and care.^30^, ^31^, ^32^, ^33^ Many changes in clinical practice have led to increased demands on physicians to document various aspects of their work, which has often been attributed as a key reason for reduced time with patients.^34^, ^35^, ^36^

The physicians believed that time pressure made it more difficult for them to maintain full focus and attention on the patient, achieve well‐functioning communication, and establish a trusting relationship with the patient, implying interdependency between many of the determinants identified in this study. Time with the patient matters; longer visits to physicians are associated with increased patient centeredness, more attention to psycho‐social problems, lower prescribing rates, better‐quality prescribing, and lower return consultation rates.^37^, ^38^ The importance of a trustful relationship between the patient and health professional for patient participation has been demonstrated in previous research.^39^, ^40^, ^41^, ^42^ Patients' motivation to participate in their own treatment and care is reduced when patients have concerns about being ignored, dismissed, not believed, or not taken seriously.^25^, ^26^, ^32^, ^41^, ^43^ A relationship that lacks trust compromises communication, because patients may limit the information they share with health professionals who they distrust or feel uncomfortable with.^44^

The physicians further believed that patient participation for safer care might be negatively affected by patients' unfamiliarity with the health care environment and a somewhat limited understanding of their own treatment and care. A study by De Brun et al^5^ also identified barriers and facilitators, noting that some patients considered safety as a “default” position, which made patient safety unimportant and irrelevant for them. Other research has shown that patients have difficulties understanding medical terminology^30^ and have insufficient knowledge about their own treatment and care.^42^ Access to information has been found to be positively associated with patients' willingness and ability to become involved in their own treatment and care.^33^, ^45^, ^46^ Information can improve self‐efficacy and risk perception, which increases the likelihood that patients can detect and speak up about errors and safety hazards.^30^

Difficulties associated with some patients' physical and mental health status constituted another barrier to patient participation in our study. Findings from several studies conducted from the patients' perspective suggest that willingness and ability to be involved in one's own treatment and care tend to be inversely proportional to the severity of the patient's disease.^47^, ^48^, ^49^, ^50^ Patients are in a vulnerable situation and put themselves under the control of health professionals because they have no choice.^4^ Poor physician‐patient communication also acted as a barrier to patient participation in our study, consistent with previous research, which has emphasized the importance of the ability to communicate well with health professionals to enable patient participation.^30^, ^41^, ^42^, ^51^, ^52^

Physicians' motivation to initiate patient engagement could act as both a barrier and a facilitator. The physicians recognized the importance of actively inviting the patient to participate. Other studies have shown that more active engagement by patients is facilitated by encouraging or instructing them to ask questions or to participate in specific actions.^28^, ^32^, ^53^ However, some physicians in our study expressed hesitancy about encouraging patients to ask questions because they saw a risk in entering into time‐consuming discussions about less important matters or discussing with patients who were never content with the replies they received. Physicians have been found to avoid discussions of certain aspects of patients' problems if they believe they cannot handle the issues well or believe they have insufficient time to do so adequately.^54^

Learning from patients who identified errors or hazards was also associated with some uncertainty on the part of the physicians. Although they recognized the relevance to patient safety of patients who detected errors and safety hazards, the physicians also admitted to feelings of guilt and embarrassment when they realized that they had made slips or mistakes that could have caused patient injuries. Physicians have been described as the “second victim” of medical errors because they can experience emotions related to sadness, fear, anger, and shame when they are involved in medical error.^44^, ^55^, ^56^, ^57^ The findings regarding physicians' ambivalent feelings towards patient participation are somewhat echoed in the study by Hrisos and Thomson^41^ where health care professionals reported that they adopted self‐protective behaviours in interactions with certain patients to distance themselves from being the target of complaints.

Although the physicians described the importance of organizational‐level routines, such as adverse event reporting, they could not point to any specific routines or tools for use in their actual meetings with patients to facilitate patient participation. These findings imply that patient participation for safer care is largely left up to the individual physician and is dependent on his or her ability and willingness to involve patients in their treatment and care. Furthermore, many of the determinants that acted as both barriers and facilitators in our study depended on individual physicians' capability to communicate well and establish a trustful relationship with their patients, and the physicians' motivation to initiate patient engagement and learn from patients who detect errors and safety hazards, which has also been found by others.^28^, ^32^, ^53^, ^58^ The dependency on the individual physician seems counterproductive to achieving more systematic patient participation of relevance to patient safety. It also contrasts with the systems perspective on patient safety widely advocated today,^59^ calling into question the extent to which patient participation can contribute to attaining safer care. We agree with The Health Foundation's summary in *Evidence scan—Involving patients in improving safety*,^58^ that a fundamental shift is needed in the way both health care professionals and patients view their roles. They described both less active strategies (eg, surveys) and more active strategies (eg, educational campaigns) to involve patients in improving safety. The most effective approach has not been investigated or determined yet, but The Health Foundation emphasizes that it is important to conduct such intervention studies.

This study has a number of shortcomings that must be considered when interpreting the results. The sample size was relatively small, although the data analysis confirmed that data saturation was reached before cessation of the interviews. The voluntary nature of participation in the study means that the physicians interviewed may differ from the broader population of physicians. The managers forwarded the invitation to all physicians in the health care facilities, and those who were interested contacted us by email or telephone. The results should not be generalized to a larger population of physicians (ie, statistical generalization). Instead, we sought analytical generalization by comparing our findings with other studies on patient participation (although most previous research was conducted from the patients' perspective). Physicians' impressions suggest the relevance of exploring potential associations between sociodemographic patient characteristics and involvement in future studies.

## 5 CONCLUSIONS

There are many barriers to patient participation to achieve safer care. Although there are also facilitators, they tend to depend on the initiatives of individual physicians and patients, because organizational‐level support may be lacking. Many of the determinants are interdependent, with physicians' perceived time constraints influencing other barriers. Patient participation depends on physicians' capability to achieve favourable physician‐patient communication and a trustful relationship with the patient. Their motivation to involve patients by initiating patient engagement and learning from patients who detect errors and safety hazards is important for achieving patient participation.

### IMPLICATIONS

This study suggests some implications. It is obvious that health care professionals need support from the health care organization to overcome barriers related to time, routines, and continuity of care. Physicians need awareness of the patients' knowledge and capability and must adapt the communication accordingly, which highlights the importance of accounting for this in physicians' training. More research is called for to study the effectiveness of different strategies to achieve patient involvement for improved patient safety.

## FUNDING

This study was funded by The Swedish Research Council for Health, Working Life and Welfare, FORTE, 2014‐4567.

## CONFLICT OF INTERESTS

The authors have declared that there is no conflict of interest.

## AUTHOR CONTRIBUTIONS

Conceptualization: Kristina Schildmeijer, Per Nilsen, Carin Ericsson, Janna Skagerström

Formal Analysis: Kristina Schildmeijer, Per Nilsen

Investigation: Kristina Schildmeijer, Carin Ericsson, Janna Skagerström

Methodology: Kristina Schildmeijer, Per Nilsen, Carin Ericsson, Janna Skagerström

Project Administration: Kristina Schildmeijer, Per Nilsen, Carin Ericsson, Janna Skagerström

Writing—Original Draft Preparation: Kristina Schildmeijer, Per Nilsen, Carin Ericsson, Anders Broström, Janna Skagerström

Writing—Review and Editing: Kristina Schildmeijer, Per Nilsen, Carin Ericsson, Anders Broström, Janna Skagerström

All authors agreed to the final version of the manuscript before submission.

## Supporting information

Supplementary Info
