# Supplementary material for: Determinants of patient participation for safer care: A qualitative study of physicians' experiences and perceptions
Source: Health Sci Rep. 2018 Sep 12;1(10):e87. doi: 10.1002/hsr2.87 (PMC6266354; doi:10.1002/hsr2.87)
Supplement: Supplementary file 1 — Supplementary Info [file HSR2-1-e87-s001.docx]

INTERVIEWGUIDE

| **Introduction questions:**  How long have you been working in this clinic?  How many years have you worked as physician?  Can you please give a short description of your work at this clinic? |
| --- |

| **1.** | According to your opinion, what can patients do to improve patient safety in their care? |
| --- | --- |

| **2.** | How can patients affect patient safety by asking questions, give their views and critique their care in conversations with health care professionals? |
| --- | --- |
| **2.1.** | Could you provide examples of such situations? |

| **3.** | Can you describe your own experiences of patients who have noticed something that have had (or could have had) significance for patient safety? |
| --- | --- |

| **4.** | How does it feel - or how would it feel **–** if a patient recognizes and addresses a mistake or risk? |
| --- | --- |

| **5.** | What difficulties might there be for the staff with patients who ask questions, have opinions on or critique their care in conversations with healthcare professionals? |
| --- | --- |

| **6.** | What do you think hinders patients to ask questions, comment on or critique their care in conversations with healthcare professionals? |
| --- | --- |

| **7.** | What facilitates patients to ask questions, comment on or critique their care in conversations with healthcare professionals? |
| --- | --- |

| **8.** | Can you tell me how you currently work to account for patients’ experiences, opinions and criticisms concerning their care? |
| --- | --- |
| **8.1.** | What routines do you have for accounting for patients’ experiences, views and criticisms? How? |

| **9.** | Can you provide suggestions on how healthcare more efficiently than today could utilize patients’ experiences, views and criticisms? |
| --- | --- |
